# Characterization of adult patients with IgG subclass deficiency and subnormal IgG2

**DOI:** 10.1371/journal.pone.0240522

**Published:** 2020-10-13

**Authors:** James C. Barton, Jackson C. Barton, Luigi F. Bertoli, Ronald T. Acton

**Affiliations:** 1 Department of Medicine, University of Alabama at Birmingham, Birmingham, Alabama, United States of America; 2 Southern Iron Disorders Center, Birmingham, Alabama, United States of America; 3 Department of Medicine, Brookwood Medical Center, Birmingham, Alabama, United States of America; 4 Department of Microbiology, University of Alabama at Birmingham, Birmingham, Alabama, United States of America; Imperial College London, UNITED KINGDOM

## Abstract

**Background:**

Adults with IgG subclass deficiency (IgGSD) with subnormal IgG2 are inadequately characterized.

**Methods:**

We retrospectively analyzed observations in unrelated adults with IgGSD evaluated in a single hematology clinic (1991–2019) and selected those with subnormal serum IgG2 (<117 mg/dL (<1.2 g/L)) without corticosteroid therapy to describe: age; prevalence of women; upper/lower respiratory infection; autoimmune condition(s); atopy; other allergy; frequent or severe respiratory tract infection in first-degree relatives; IgG, IgG subclasses, IgA, and IgM; blood lymphocyte subpopulations; human leukocyte antigen (HLA)-A and -B types and haplotypes; and 23-valent pneumococcal polysaccharide vaccination (PPSV23) responses. We determined the prevalence of subnormal IgG2 among unrelated adults with IgGSD without corticosteroid therapy and compared general characteristics of those with and without subnormal IgG2.

**Results:**

There were 18 patients (94.4% women) with subnormal IgG2. Mean age was 52 ± 11 y. Upper/lower respiratory infection occurred in 94.4%/74.8%, respectively. Autoimmune condition(s), atopy, other allergy, and frequent or severe respiratory infection in first-degree relatives occurred in 44.4%, 44.4%, 61.1%, and 22.2%, respectively. Median IgG2 was 105 mg/dL (83, 116). Subnormal IgG, IgG1, IgG3, IgG4, IgA, and IgM was observed in 66.7%, 50.0%, 100.0%, 5.6%, 33.3%, and 0%, respectively. Lymphocyte subpopulations were normal in most patients. HLA frequencies were similar in patients and controls. Three of 4 patients had no protective *S*. *pneumoniae* serotype-specific IgG levels before or after PPSV23. These 18 patients represent 7.6% of 236 adults with IgGSD. Prevalence of subnormal IgG, subnormal IgG3, and subnormal IgA was significantly greater in 18 adults with subnormal IgG2 than 218 adults without subnormal IgG2. Prevalence of subnormal IgM was significantly lower in patients with subnormal IgG2.

**Conclusions:**

Characteristics of adults with IgGSD with subnormal IgG2 include female predominance, other immunologic abnormalities, subnormal IgG3 and/or IgG1, lack of HLA-A and -B association, and suboptimal PPSV23 response.

## Introduction

Four Ig (immunoglobulin) G (IgG) subclasses occur in humans and are numbered in order of their decreasing abundance in plasma or serum [1]. In normal adults, IgG2 represents ~one-third of serum IgG [1,2]. IgG2 activates complement less readily than IgG1 and IgG3 [1], has low affinity for Fc receptors on phagocytes (FcγRII) [1], and crosses the placenta less freely than other IgG subclasses [1,3,4]. The half-life of IgG2 is ~21 d, similar to that of IgG1 and IgG4 [5]. Median IgG2 levels in healthy men and women ages 18–71 y do not differ significantly [6]. IgG2 is the predominant antibody that responds to bacterial polysaccharide antigens [7–9]. Some adults with frequent or severe respiratory infection due to encapsulated bacteria have suboptimal IgG2 responses to polysaccharide antigens [7,8,10]. Some adults with subnormal or absent IgG2 have recurrent or chronic respiratory tract infection [11–13]. Other adults with subnormal or absent IgG2 are healthy [14–18].

IgG subclass deficiency (IgGSD), a heterogeneous subtype of primary immunodeficiency, is defined as the triad of frequent or severe respiratory tract infection, one or more subnormal IgG subclass level(s) (≤2 standard deviations below respective means) unexplained by other causes, and decreased IgG response to pneumococcal polysaccharides [19–23]. Many adults with IgGSD also have autoimmune condition(s) or atopy [21,24–26]. IgGSD occurs in ~1 in 10,000 persons [27]. In children, IgG2 deficiency is the most common subtype of IgGSD [27]. Our informal experience suggested that subnormal IgG2 is uncommon in adults with IgGSD. We sought to characterize adult patients with IgGSD whose IgG2 levels were subnormal, alone or in combination with other subnormal Ig levels.

To learn more about adults with IgGSD immunophenotypes that include subnormal IgG2, we performed a retrospective chart review to identify such patients diagnosed and characterized in a hematology clinic and selected those with subnormal IgG2 for further analysis. All patients were evaluated and diagnosed to have IgGSD during the interval November 1991-December 2019 using the same criteria. We analyzed: age at diagnosis; prevalence of women; upper and lower respiratory tract infection; other infection; autoimmune condition(s); atopy; other allergy; reports of frequent or severe respiratory tract infection in first-degree relatives; serum levels of IgG, IgG subclasses, IgA, and IgM; blood lymphocyte subpopulations; human leukocyte antigen (HLA)-A and -B types and haplotypes; and responses to 23-valent pneumococcal polysaccharide vaccination (PPSV23; Pneumovax®23, pneumococcal vaccine polyvalent (Merck and Co., Inc., Kenilworth, NJ)). We also determined the prevalence of the present patients with subnormal IgG2 among unrelated adults diagnosed to have IgGSD without corticosteroid therapy reports in this clinic during the interval 1991–2019 and compared general characteristics of those with and without subnormal IgG2. We discuss characteristics of the present patients, attributes of other adult cohorts with and without IgGSD pertinent to IgG2, and factors that influence IgG2 levels.

## Methods

### Ethical approval

This work was performed according to the principles of the Declaration of Helsinki [28]. Western Institutional Review Board provided an exemption under 45 CFR 46.101(b)(4) pertinent to this study on 18 October 2018 (submission 2535878–44170911; 2 October 2018). Obtaining informed consent was not required because this study involved retrospective chart review and analyses of observations recorded in routine medical care and does not include personal identifier information.

### Patient selection

We reviewed the medical records of consecutive, unrelated, self-identified non-Hispanic white adults (ages ≥18 y) referred to a single outpatient hematology clinic (Southern Hematology & Oncology, P.C., Birmingham, AL, USA) because they had frequent or severe upper or lower respiratory tract infection inadequately managed with antibiotic and ancillary therapy, were subsequently diagnosed to have IgGSD, underwent HLA typing and haplotyping, and had no first-degree relatives with IgGSD or other Ig deficiency previously known to this clinic [21,29]. Frequent respiratory tract infection was defined as four or more episodes per year that required antibiotic therapy. Severe respiratory tract infection was defined as any respiratory tract infection that required in-hospital treatment [30].

IgGSD is defined as the triad of frequent or severe respiratory tract infection, one or more subnormal IgG subclass level(s) (≤2 standard deviations below respective means) unexplained by other causes, and decreased IgG response to pneumococcal polysaccharides [19–23]. Patients selected for this study had subnormal IgG2, alone or in combination with other subnormal Ig levels. All patients were evaluated and diagnosed to have IgGSD during the interval November 1991-December 2019.

### General characteristics

Upper respiratory tract infection was defined as reports of sinusitis, otitis media, mastoiditis, pharyngitis, and tonsillitis. Lower respiratory tract infection was defined as reports of bronchitis, pneumonia, bronchiectasis, and pleurisy. Frequent respiratory tract infection was defined as four or more episodes per year that required antibiotic therapy. Severe respiratory tract infection was defined as any respiratory tract infection that required in-hospital treatment [30]. We defined other infections as those in sites other than upper or lower respiratory tract [31].

We compiled reports of duration of frequent or severe respiratory tract infection before IgGSD diagnosis. Age at onset of frequent or severe respiratory tract infection was defined as the difference between age at IgGSD diagnosis and reported duration of frequent or severe respiratory tract infection before IgGSD diagnosis [30].

Autoimmune condition(s) and atopy (allergic asthma, allergic rhinitis, and allergic dermatitis/eczema) were diagnosed by referring physicians. Other allergy manifestations included urticaria, angioedema, or anaphylaxis that occurred with or without exposure to specific medications, foods, or environmental allergens [32].

### Prevalence of IgGSD with subnormal IgG2

We used office computer software and manual chart review to determine the number of all unrelated adults evaluated and diagnosed to have IgGSD in this clinic, regardless of IgG subclass immunophenotype, during the interval July 2002-December 2019. We also included adults with IgGSD described in a report representing the interval November 1991-June 2002 [32]. Subnormal IgG4 level alone was not a sufficient criterion for IgGSD diagnosis. We excluded adults who reported corticosteroid therapy. We compared the number of unrelated adults with IgGSD immunophenotypes that included subnormal IgG2 with the number of all unrelated adults evaluated and diagnosed to have IgGSD in this clinic during the interval November 1991-December 2019 who reported no corticosteroid therapy.

### Patient exclusions

We excluded patients with: reports of corticosteroid therapy; absence of IgG2 and other immunophenotype abnormalities typical of persons with deletion of multiple immunoglobulin heavy chain constant regions (chromosome 14q32.33) [14,16] or a more localized deletion of *IGHG2* affecting heavy chain constant region gamma-2 [33]; common variable immunodeficiency; hypogammaglobulinemia due to B-cell neoplasms, organ transplantation, immunosuppressive therapy, anti-cancer chemotherapy, or increased Ig loss; malignancy; monoclonal gammopathy; infection with parasites, *Mycobacterium* sp., human immunodeficiency virus (HIV), Epstein-Barr virus, or cytomegalovirus; allergic bronchopulmonary aspergillosis; eosinophilic granulomatosis with polyangiitis (Churg-Strauss); ataxia-telangiectasia or other syndromic disorders; rare immunodeficiency syndromes associated with elevated serum IgE levels; and incomplete evaluations. The present study design did not include reviewing medical records of or otherwise recruiting control subjects from general clinic adult populations.

### Laboratory methods

IgG and IgG subclasses at diagnosis were measured using rate nephelometry (Laboratory Corporation of America, Burlington, NC, USA) before IgG replacement therapy was initiated. We defined mean ± 2 standard deviations (SD) as reference limits [32,34]: IgG 700–1600 mg/dL (7.0–16.0 g/L); IgG1 422–1292 mg/dL (4.2–12.9 g/L); IgG2 117–747 mg/dL (1.2–7.5 g/L); IgG3 41–129 mg/dL (0.4–1.3 g/L); IgG4 1–291 mg/dL (0–2.9 g/L); IgA 70–400 mg/dL (700–4000 mg/L); and IgM 40–230 mg/dL (400–2300 mg/L). Reference ranges for untreated patients with IgGSD did not change during the interval 1991–2019. Subnormal Ig levels were defined as those below the corresponding lower reference limits. Subnormal Ig levels were documented twice in all adults at times they did not have acute infection. We used the second IgG subclass values for the present analyses.

Blood lymphocyte subpopulations were measured using flow cytometry (Laboratory Corporation of America, Burlington, NC, USA). Reference ranges (mean ± 2 SD) are: CD19+ B-lymphocytes 12–645 cells/μL; CD3+/CD4+ T-lymphocytes 359–1,519 cells/μL; CD3+/CD8+ T-lymphocytes 109–897 cells/μL; and CD56+/CD16+ natural killer cells 24–406 cells/μL. Reference ranges did not change during the interval 1991–2019. Subnormal levels and elevated levels were defined as those below and above the corresponding lower reference limits, respectively.

We analyzed data from 1,321 unrelated Alabama non-Hispanic whites who had undergone HLA-A and -B typing as part of paternity testing. HLA-A or -B type positivity, defined as the occurrence of either one or two of the same HLA-A or -B types, respectively, was calculated using observations from unrelated men and women regardless of whether the man was determined to be the father of the child in question or not. Thus, there were more men than women [32,35,36]. We ascertained HLA-A and -B haplotypes in 751 unrelated Alabama non-Hispanic whites who had undergone testing to establish paternity by assessing HLA-A and -B types in children and their respective mothers or putative fathers. An equal number of men and women were used to ascertain haplotypes [32,35]. Frequencies of major HLA-A and -B haplotypes observed in controls were similar to those of Caucasians enrolled in a national bone marrow donor program [37].

*Streptococcus pneumoniae* serotype-specific IgG antibodies were measured by clinical laboratories (Laboratory Corporation of America, Burlington, NC, and ViraCor-IBT, Lee's Summit, MO). Diluents for patient samples tested for *S*. *pneumoniae* serotype-specific IgG contained C-polysaccharide and polysaccharide type 22. No patient received IgG replacement therapy before IgG test panels were reported. Responses to 23-valent pneumococcal polysaccharide vaccination (PPSV23; Pneumovax®23) were measured as *S*. *pneumoniae* serotype-specific IgG antibody levels before vaccination and again ≥28 d after vaccination (post-PPSV23 antibody panels). All measurements were performed before IgG replacement therapy was initiated. Test panels included measurements of antibodies specific for 6, 7, or 14 serotypes as described previously [38]. We defined serotype-specific IgG levels as either protective (≥1.3 mg/L) or non-protective (<1.3 mg/L) [39]. We defined aggregate pneumococcal polysaccharide response as *S*. *pneumoniae* serotype-specific IgG >1.3 μg/mL for ≥70% of serotypes in specimens obtained ≥28 d after PPSV23 [40].

### Statistics

The analytic data set consisted of observations on 18 patients with and 218 patients without subnormal IgG2. All age-at-diagnosis, duration-of-infection, and age-at-onset of infection data were expressed to the nearest whole year. IgG4 levels <1 mg/dL were imputed as 0.5 mg/dL. We evaluated continuous data for normality using d'Agostino's and Shapiro-Wilk tests. Descriptive data are displayed as enumerations, percentages, medians (range), or means (± 1 standard deviation (SD)). Age data were normally distributed and thus mean age values were compared using Student's t test (two-tailed). Differences in median values of IgG2 were compared using the Mann-Whitney U test. We computed the confidence interval (with continuity correction) of one proportion. We computed positivity for HLA-A and -B types (18 patients) and frequency of HLA-A and -B haplotypes (17 patients) and compared them to those of controls (1,321 for most types; 751 for most haplotypes). We compared differences in proportions of dichotomous variables using Fisher's exact test (two-tailed).

We performed a backward stepwise regression on IgG2 levels using these independent variables in 18 patients with subnormal IgG2: age at diagnosis; sex; upper and lower respiratory tract infection; other infection; autoimmune condition(s); atopy; other allergy; reports of frequent or severe respiratory tract infection in first-degree relatives (dichotomous); and IgG, IgG1, IgG3, IgG4, IgA, and IgM levels.

We defined values of *p* <0.05 to be significant. Bonferroni corrections were applied to control type I error rates at 0.05 for separate comparisons of dichotomous HLA type and haplotype data. For univariate comparisons of general characteristics of adults with and without subnormal IgG2, we did not use a Bonferroni "correction" because most data were not positively correlated and we did not wish to produce "false negative" results [41]. Analyses were performed with Excel 2000^®^ (Microsoft Corp., Redmond, WA, USA) and GB-Stat^®^ (v. 10.0, 2003, Dynamic Microsystems, Inc., Silver Spring, MD, USA).

## Results

### Prevalence of subnormal IgG2 in adults with IgGSD

We identified 360 unrelated adult patients diagnosed to have IgGSD during the interval November 1991-December 2019 (126 from November 1991-June 2002 [32] and 234 from July 2002-December 2019). Of these 360 patients, 236 reported no corticosteroid therapy. The present 18 patients with subnormal IgG2 represent 7.6% of the 236 patients [95% confidence interval: 4.7, 12.0] with IgGSD immunophenotypes without reports of corticosteroid therapy.

### General characteristics of 18 patients with IgGSD and subnormal IgG2

There were 17 women (94.4%) and one man (5.6%). The mean age at diagnosis of all patients was 52 ± 11 y (Table 1). Upper and lower respiratory tract infection interpreted as bacterial was reported by 94.4% and 77.8% of patients, respectively. Thirteen patients (72.2%) reported having both upper and lower respiratory tract infection.

**Table 1:** Characteristics of adults with IgGSD with and without subnormal IgG2 (<117 mg/dL)^a^.

Characteristic	Subnormal IgG2 (n = 18)	No subnormal IgG2 (n = 218)	Value of p
Women, % (n)	94.4 (17)	79.4 (173)	0.2110
Mean age, y (± 1 SD)	52 ± 11	50 ± 13	0.4455
Upper respiratory tract infection, % (n)	94.4 (17)	93.6 (204)	~1.0000
Lower respiratory tract infection, % (n)	77.8 (14)	81.7 (178)	0.7522
Upper and lower respiratory tract infection, % (n)	72.2 (13)	77.1 (168)	0.5761
Autoimmune condition(s), % (n)	44.4 (8)^b^	47.2 (103)^c^	~1.0000
Atopy, % (n)	44.4 (8)^d^	28.0 (61)^e^	0.1767
Other allergy, % (n)	61.1 (11)	45.2 (99)	0.2260
Frequent/severe respiratory tract infection in first-degree relatives, % (n)	22.2 (4)	40.8 (89)	0.1391
Subnormal IgG (<700 mg/dL), % (n)	66.7 (12)	37.2 (81)	0.0218
Subnormal IgG1 (<422 mg/dL), % (n)	50.0 (9)	68.3 (149)	0.1235
Median IgG2, mg/dL (range)	105 (83, 116)	257 (117, 644)	<0.0001
Subnormal IgG3 (<41 mg/dL), % (n)	94.4 (17)	67.4 (147)	0.0155
Subnormal IgG4 (<1 mg/dL), % (n)	5.6 (1)	5.0 (11)	~1.0000
Subnormal IgA (<70 mg/dL)	33.3 (6)^f^	4.1 (9)^g^	0.0003
Subnormal IgM (<40 mg/dL)	0	23.4 (51)	0.0156

^a^ Abbreviations: IgGSD, immunoglobulin G subclass deficiency; IgG1, immunoglobulin G1; IgG2, immunoglobulin G2; IgG3, immunoglobulin G3; IgG4, immunoglobulin G4; SD, standard deviation. No patient had monoclonal gammopathy detected by serum electrophoresis and immunofixation.

^b^ Eight patients (44.4%) had an autoimmune condition(s): rheumatoid arthritis (4); autoimmune/Hashimoto thyroiditis (3); lupus (2); Sjögren syndrome (2); anticardiolipin antibody syndrome (1); and positive anti-nuclear antibody ≥1:80 without greater specificity (1). Three patients (16.7%) had two or more autoimmune conditions.

^c^ One hundred and three patients (47.2%) had an autoimmune condition(s): autoimmune/Hashimoto thyroiditis (39); rheumatoid arthritis (20); Sjögren syndrome (20); systemic lupus erythematosus (20); inflammatory arthritis not otherwise specified (6); psoriatic arthritis (6); ankylosing spondylitis (5); Graves disease (4); psoriasis (4); Raynaud phenomenon (4); Crohn disease (3); pernicious anemia (3); positive anti-nuclear antibody ≥1:80 without greater specificity (3); undifferentiated connective tissue disorder (3); autoimmune diabetes (2); Behçet disease (2); multiple sclerosis (2); ulcerative colitis (2); anti-cardiolipin antibody syndrome (1); autoimmune hemolytic anemia (1); autoimmune uveitis (1); glutamic acid decarboxylase autoantibody (1); Guillain-Barré syndrome (1); myasthenia gravis (1); and polymyalgia rheumatica (1). Forty-two patients (19.3%) had two or more autoimmune conditions.

^d^ Seven patients had allergic asthma. One other patient had allergic rhinitis.

^e^ Sixty-one patients had atopy (52 allergic asthma; 15 allergic rhinitis; 3 allergic dermatitis). Each of eight patients had two (or three) subtypes of atopy.

^f^ The lowest IgA level observed in this subgroup was 32 mg/dL. Four of these 18 patients had both subnormal IgG and subnormal IgA.

^g^ The lowest IgA level observed in this subgroup was 3 mg/dL. None of these 218 patients had both subnormal IgG and subnormal IgA.

Median duration of frequent or severe respiratory tract infection before IgGSD diagnosis was 13 y (1, 39). Median age at onset of frequent or severe infection was 40 y (15, 61). One patient (5.6%) reported having frequent or severe respiratory tract infection at age <18 y.

Eight patients (44.4%) reported infection in sites other than upper or lower respiratory tract. Some patients reported more than one other infection. Of these eight patients, four reported non-invasive oral or vaginal yeast infection (50.0%) attributed to *Candida* sp., four reported bacterial infection of skin or nail beds (50.0%), two reported bacterial urinary tract infection (25.0%), and two reported bacterial infection of gingivae or teeth (25.5%). One patient reported having herpes zoster (5.6%). One patient (5.6%): reported having had sepsis.

Eight patients (44.4%) had autoimmune condition(s) (Table 1). Eight patients (44.4%) had atopy (Table 1). Eleven patients (61.1%) had other allergy. Four patients (22.2%) reported that they had one or more first-degree relatives with frequent or severe respiratory tract infection (Table 1).

### Comparison of adults with and without subnormal IgG2

The median IgG2 value in 18 adults with IgGSD and subnormal IgG2 was significantly lower than that of 218 adults with IgGSD without subnormal IgG2 (by definition) (Table 1). The prevalence of subnormal IgG, subnormal IgG3, and subnormal IgA was significantly greater in adults with than without subnormal IgG2 (Table 1). The prevalence of subnormal IgM was significantly lower in adults with than without subnormal IgG2 (Table 1).

### IgG subclass immunophenotypes

Each of 18 patients had subnormal IgG2, by definition. IgG2 represented 19.5 ± 5.6% of the sum of IgG subclasses. Median IgG2 was 106 mg/dL (83, 117). Subnormal IgG, subnormal IgG1, subnormal IgG3, and subnormal IgG4 was observed in 66.7%, 50.0%, 100.0%, and 5.6% of patients, respectively (Table 2).

**Table 2:** Ig immunophenotypes in 18 adult patients with IgGSD and subnormal IgG2^a^.

Subnormal IgG subclass phenotype	Percent of patients (n)
G1/G2/G3	44.4 (8)
G2/G3	50.0 (9)
G1/G2/G3/G4	5.6 (1)
Total	100.0 (18)

^a^ Abbreviations: IgGSD, immunoglobulin G subclass deficiency; IgG, immunoglobulin G.

### Regression on IgG2 levels in 18 adults with IgGSD and subnormal IgG2

We performed a backward stepwise regression on IgG2 levels using these independent variables: age at diagnosis; sex; upper and lower respiratory tract infection; other infection; autoimmune condition(s); atopy; other allergy; reports of frequent or severe respiratory tract infection in first-degree relatives (dichotomous); and IgG, IgG1, IgG3, IgG4, IgA, and IgM levels. This revealed a single inverse association: other allergy (p = 0.0440; analysis of variance p = 0.0440). This regression accounted for 23.0% of the variance of IgG2.

### Blood lymphocyte subpopulations

Flow cytometry measurements were available in 16 patients with IgGSD and subnormal IgG2 (81.6%) (Table 3). CD19+ B-lymphocyte counts were within the reference range for all patients. CD4+ T-lymphocyte counts were elevated in two patients (12.5%) and subnormal in one patient (6.3%). CD8+ T-lymphocyte and CD16+/CD56+ NK lymphocyte counts were within the respective reference ranges for all patients (Table 3).

**Table 3:** Blood lymphocyte subpopulations in 16 adults with IgGSD and subnormal IgG2^a^.

Lymphocyte subpopulation	Adults (n = 16)
Median CD19+ B-cells/μL (range)	248 (66, 626)
Subnormal CD19+ B-cells, % (n)	0
Elevated CD19+ B-cells, % (n)	0
Median CD4+ T-cells/μL (range)	962 (57, 2271)
Subnormal CD4+ T-cells, % (n)	6.3 (1)
Elevated CD4+ T-cells, % (n)	12.5 (2)
Median CD8+ T-cells/μL (range)	250 (111, 879)
Subnormal CD8+ T-cells, % (n)	0
Elevated CD8+ T-cells, % (n)	0
Median CD56+/CD16+ NK cells/μL (range)	125 (35, 284)
Subnormal CD56+/CD16+ NK cells, % (n)	0
Elevated CD56+/CD16+ NK cells, % (n)	0

^a^ Abbreviations: IgGSD = Immunoglobulin G subclass deficiency; IgG2, immunoglobulin G2; NK, natural killer. Flow cytometry measurements were available in 16 patients (88.9%). Reference ranges (mean ± 2 SD) are: CD19+ B-lymphocytes 12–645 cells/*μ*L; CD3+/CD4+ T-lymphocytes 359–1,519 cells/*μ*L; CD3+/CD8+ T-lymphocytes 109–897 cells/*μ*L; and CD56+/CD16+ NK cells 24–406 cells/*μ*L. Levels are expressed as median (range). Subnormal levels and elevated levels were defined as those below and above the corresponding lower reference limits, respectively.

### HLA-A and -B phenotypes and haplotypes

Nine HLA-A phenotypes were identified in 18 patients with IgGSD and subnormal IgG2. Phenotype positivities for HLA-A*02, -A*01, and -A*03 were highest (S1 Table). Phenotype frequencies did not differ significantly between patients and 1,321 unrelated Alabama non-Hispanic whites who had undergone paternity testing. (S1 Table). Thirteen HLA-B phenotypes were identified in 18 patients. Phenotype frequencies of HLA-B*07 and -B*08 were highest (S2 Table). There were no significant differences between phenotype frequencies in patients and 1,321 unrelated Alabama non-Hispanic whites who had undergone paternity testing after Bonferroni correction (S2 Table). Frequencies of 15 haplotypes in 17 patients with IgGSD and 751 unrelated Alabama non-Hispanic whites who had undergone testing to establish paternity did not differ significantly after Bonferroni correction (S3 Table).

### *S*. *pneumoniae* serotype-specific IgG

Pre- and post-PPSV23 *S*. *pneumoniae* IgG serotype-specific antibody levels were available in four of 18 patients with subnormal IgG2 (22.2%). Three of these four patients had no protective serotype-specific antibody levels either before or after PPSV23. The fourth patient had 0/6 protective serotype-specific antibody levels pre-PPSV23 and 5/6 protective serotype-specific antibody levels post-PPSV23. Thus, three of these four patients with subnormal IgG2 had no aggregate IgG response to PPSV23 [40]. Data available for the remaining 14 patients were insufficient to determine aggregate PPSV23 response.

## Discussion

The present study characterizes 18 adult patients with IgGSD whose subnormal IgG subclasses included IgG2. General characteristics of this cohort are similar to those of cohorts of adults with IgGSD without subnormal IgG2, including: age at diagnosis [25,26,31,38,42]; predominance of women [26,31,38,42]; duration of frequent or severe respiratory tract infection and age of onset of frequent or severe respiratory tract infection before diagnosis of IgGSD [30,42]; other infection [31]; high proportions of subjects with autoimmune condition(s), atopy, or other allergy [25,26,31,38]; reports of first-degree relatives who have frequent or severe infection [25,26,31]; and normal blood lymphocyte subpopulation levels in most patients [23,26,31,38].

IgG2 as a proportion of IgG subclasses was 20% in the present patients, of whom 67% had subnormal IgG. In normal adults, IgG2 represents ~one-third of serum IgG [1,2]. These observations indicate that both relative and absolute levels of IgG2 are subnormal in the present patients.

Subnormal IgG2 is relatively common in healthy adults without frequent or severe respiratory tract infection or related abnormalities and does not indicate that such persons have immune deficiency. In 8015 Missouri adult blood donors (96% white, 54% men), log IgG2 levels were not normally distributed [18]. The prevalence of donors with IgG2 levels 2–3 SD and >3 SD below the mean was 1.6-fold and 1.5-fold more than predicted, respectively [18]. Further study of these donors without a history of recurrent infection who had subnormal IgG2 levels revealed that the "normal" range for those with G2m(23)- allotype (a IgG2 heavy-chain allele) was 35% lower than for those with G2m(23)+ allotype [43]. Some donors with G2m(23)- allotype and IgG2 levels <0.8 g/L had other abnormalities, including subnormal IgA and/or IgG4 levels and decreased IgG2 expression *in vitro* [43]. In 65 German adult blood donors without recurrent infection or elevated C-reactive protein levels, two had IgG2 levels >2 SD below the mean [34]. Accordingly, the yield of measuring serum IgG2 to identify adults in general populations who have decreased responsiveness to bacterial polysaccharides or increased susceptibility to respiratory tract infection would be limited.

Subnormal IgG1, subnormal IgG3, and subnormal IgG4 occurred in 50%, 100%, and 6% of the present 18 patients, respectively, although neither IgG1, IgG3, nor IgG4 levels were significantly associated with IgG2 levels, after adjustment for other variables. Selective subnormal IgG1 [25,31], selective subnormal IgG3 [23,26,38], and selective subnormal IgG4 [23,44] probably contribute to susceptibility to frequent or severe respiratory tract infection in adults with IgGSD. Subnormal IgG1 [34,45], subnormal IgG3 [34], and subnormal IgG4 [18,34] also occur in healthy adults without frequent or severe respiratory tract infection.

Subnormal IgA and IgM levels occurred in 33% and 0% of the present 18 patients, respectively, although IgA and IgM levels were not associated with IgG2 levels, after adjustment for other variables. Although IgA was detectable in the serum of all of the present patients, it is plausible but unproven that subnormal IgA levels contributed to increased susceptibility of respiratory tract or other infection [46–48].

In this study, the prevalence of subnormal IgG, subnormal IgG3, and subnormal IgA was significantly greater in 18 adults with subnormal IgG2 than corresponding values in 218 adults without subnormal IgG2. Subnormal IgG2 and subnormal IgG3 could partly account for subnormal IgG. Subnormal IgG2 was associated with increased infection risk in some persons with subnormal IgA [49], occurred with subnormal IgA and subnormal IgE in a healthy blood donor [15], and is linked to subnormal IgA in a chromosome 6p haplotype in some kinships [50]. Subnormal IgG3 is linked to a chromosome 6p SNP haplotype in adults with hemochromatosis and *HFE* p.C282Y homozygosity [51].

Blood levels of CD19+ B-lymphocyte, CD4+ T-lymphocyte, CD8+ T-lymphocyte, and CD56+/CD16+ NK lymphocyte subpopulations were normal in most of the present 16 patients we studied, consistent with observations in cohorts of adults with IgGSD whose subnormal IgG subclasses did not include IgG2 [23,26,31,38]. It is plausible but unproven that some lymphocyte subpopulation values outside respective reference ranges in the present patients were due to age [52], sex [52], or autoimmune condition(s) [53–55].

The prevalence of HLA-A and -B types and haplotypes in the present 18 patients with subnormal IgG2 and population controls was similar. Subnormal IgG3 occurred in 100% of the present patients. Taken together, these observations imply that a locus that modulates levels of IgG2 and IgG3 in the present patients is not linked to the chromosome 6p region that includes HLA-A and -B. It is possible that subnormal IgG2 in some of the present patients is due to a heritable or acquired mechanism unrelated to those that cause subnormal IgG3 only, subnormal IgG1 only, or subnormal IgG1/IgG3 only.

Four of the present 18 patients were tested with both pre- and post-PPSV23 *S*. *pneumoniae* serotype-specific antibody panels. No protective levels of IgG specific for *S*. *pneumoniae* serotypes before or after PPSV23 were detected in three of the four patients. Non-protective levels of IgG specific for diverse *S*. *pneumoniae* serotypes before or after PPSV23 were common in adults with IgGSD and normal IgG2 levels [23,26,31,38]. In one study, serotype-specific IgG responses to Pneumovax®23 were greater in adults with IgGSD and subnormal IgG1 only than subnormal IgG3 only [40]. Some adults with frequent or severe respiratory tract infection due to encapsulated bacteria have suboptimal IgG2 responses to polysaccharide antigens [7,8,10]. The median pre-PPSV23 IgG2 concentration was significantly lower in adults referred for immunological evaluation because they had frequent respiratory tract infection than in healthy controls [10]. In adults with IgA deficiency, susceptibility to respiratory tract infection was related to subnormal IgG2, subnormal IgG4, and low serum concentrations of pneumococcal polysaccharide antibodies [56]. Among 15 healthy adult blood donors with subnormal IgG2, 14 (93%) had PPSV23 responses similar to those of control subjects [43].

The present 18 patients with subnormal IgG2 (<117 mg/dL) represent 7.6% of 236 unrelated Alabama adults with IgGSD immunophenotypes without corticosteroid therapy reports. In 100 English adults with chronic or recurrent respiratory tract infection, seven (7.0%) had serum IgG2 levels <3 SD below the reference range [13]. In 59 Korean adults with IgGSD and chronic airway disease, one (1.7%) had subnormal IgG2 (<241.8 mg/dL) [22]. In a California IgGSD cohort, 21/78 adults (26.9%) had subnormal IgG2 (<237 mg/dL), alone or in combination with other subnormal IgG subclass levels [23]. Referral differences and between-laboratory variation in methods of analysis, control data, and consequent IgG2 reference limits could partly account for these prevalence differences. Regardless, these observations suggest that adults with IgGSD whose subnormal IgG subclasses include IgG2 occur in diverse race/ethnicity groups and their proportions in case series of adults with IgGSD are relatively low.

Four of the present 18 patients (22%) had both subnormal IgG and subnormal IgA. Based solely on their respective histories of infection and exclusion of secondary causes of subnormal Ig levels, these patients would be classified to have common variable immunodeficiency (CVID) [57,58]. Characterization of IgG responses to *S*. *pneumoniae* capsular polysaccharides before and after PPSV23 were not available in these cases, nor was assessment of isohemagglutinins or the proportion of switched memory B-lymphocytes. Accordingly, it is unknown whether these four patients would eventually develop or be diagnosed to have CVID. In a study in which characteristics of adults with IgGSD and CVID were compared, patients with CVID had lower IgG and IgA levels (by definition) and a greater prevalence of autoimmune conditions [21].

This study has other uncertainties and limitations. The number of adults with IgGSD we identified to have subnormal IgG2 was low and represents a small proportion of a relatively large IgGSD cohort. Thus, comparing attributes of subgroups of the present cohort was not appropriate statistically. Identification of bacteria or other agents that caused infection in most of the present patients was not available. Study of greater numbers of adult patients with IgGSD whose subnormal IgG subclasses included IgG2 may have permitted a demonstration of significant differences between HLA-A and -B type positivities and haplotype frequencies, if such differences exist. Characterizing first-degree relatives of the present patients with frequent or severe respiratory tract infection, studying the effects of IgG replacement therapy in the present patients, and performing analyses to identify mutations in *NFKB2*, *CXCR4*, *TACI*, *CTLA4*, *STAT*, *PI3KCD*, or other genes were beyond the scope of this study. The relationship of subnormal IgG2 in the present patients to subnormal IgG2 described in children with respiratory tract infection [59] is unknown.

## Conclusions

We conclude that characteristics of adults with IgGSD with subnormal IgG2 include female predominance, other immunologic abnormalities, subnormal IgG3 and/or IgG1, lack of HLA-A and -B association, and suboptimal PPSV23 response.

## Supporting information

S1 Data Observations in 236 adults with IgGSD (18 with and 218 without subnormal IgG2). (XLSX)

S1 Table HLA-A phenotype frequencies in 18 adults with IgGSD and subnormal IgG2 and 1,321 control individuals. (DOCX)

S2 Table HLA-B phenotype frequencies in 18 adults with IgGSD and subnormal IgG2 and 1,321 control individuals. (DOCX)

S3 Table HLA-A and -B haplotype frequencies in 17 adults with IgGSD and subnormal IgG2 and 751 control individuals. (DOCX)
