# Supplementary material for: Characterization of adult patients with IgG subclass deficiency and subnormal IgG2
Source: PLoS One. 2020 Oct 13;15(10):e0240522. doi: 10.1371/journal.pone.0240522 (PMC7553271; doi:10.1371/journal.pone.0240522)
Supplement: S1 Table — (DOCX) [file pone.0240522.s002.docx]

**S1 Table.** HLA-A phenotype frequencies in 18 adults with IgGSD.^a^

| **HLA-A phenotype** | **Patient frequency (n)** | **Controls frequency (n)** | **Value of p** |
| --- | --- | --- | --- |
| A*01 | 0.3333 (6) | 0.3397 (448/1,319) | 1.0000 |
| A*02 | 0.6111 (11) | 0.5206 (682/1,310) | 0.4854 |
| A*03 | 0.2778 (5) | 0.2739 (361/1,318) | 1.0000 |
| A*11 | 0.0556 (1) | 0.1136 (150/1,320) | 0.7112 |
| A*23 | 0.0556 (1) | 0.0375 (47/1,254) | 0.5020 |
| A*24 | 0.0222 (4) | 0.1304 (165/1,265) | 0.2814 |
| A*25 | 0.0556 (1) | 0.0297 (38/1,281) | 0.4244 |
| A*26 | 0 | 0.0634 (81/1,278) | 0.6221 |
| A*28 | 0 | 0.0773 (102/1,320) | 0.6326 |
| A*29 | 0.1667 (3) | 0.0620 (80/1,290) | 0.1009 |
| A*30 | 0 | 0.0405 (53/1,308) | 1.0000 |
| A*31 | 0 | 0.0350 (44/1,258) | 1.0000 |
| A*32 | 0 | 0.0536 (66/1,232) | 0.6192 |
| A*33 | 0 | 0.0176 (22/1,248) | 1.0000 |
| A*34 | 0.0556 (1) | 0.0044 (5/1,146) | 0.0895 |

^a^ Abbreviations: HLA, human leukocyte antigen; IgGSD, immunoglobulin G subclass deficiency.
