# Supplementary material for: Characterization of adult patients with IgG subclass deficiency and subnormal IgG2
Source: PLoS One. 2020 Oct 13;15(10):e0240522. doi: 10.1371/journal.pone.0240522 (PMC7553271; doi:10.1371/journal.pone.0240522)
Supplement: S2 Table — (DOCX) [file pone.0240522.s003.docx]

**S2 Table.** HLA-B phenotype frequencies in 18 adults with IgGSD.^a^

| **HLA-B phenotype** | **Patient frequency (18)** | **Controls frequency (n)** | **Value of p**^b^ |
| --- | --- | --- | --- |
| B*07 | 0.5556 (10) | 0.2259 (298/1,321) | 0.0026 |
| B*08 | 0.3333 (6) | 0.2487 (327/1,315) | 0.4149 |
| B*13 | 0 | 0.0343 (45/1,269) | 1.0000 |
| B*14 | 0.0556 (1) | 0.0639 (84/1,314) | 1.0000 |
| B*15 | 0.1667 (3) | 0.0144 (19/1,320) | 0.0027 |
| B*18 | 0.1667 (3) | 0.0775 (97/1,251) | 0.1637 |
| B*27 | 0.1111 (2) | 0.0895 (118/1,318) | 0.6727 |
| B*35 | 0.1111 (2) | 0.1406 (184/1,309) | 1.0000 |
| B*37 | 0 | 0.0240 (28/1,167) | 1.0000 |
| B*38 | 0 | 0.0193 (23/1,194) | 1.0000 |
| B*39 | 0.0556 (1) | 0.0225 (27/1,198) | 0.3445 |
| B*40 | 0.1667 (3) | 0.0265 (35/1,321) | 0.0128 |
| B*41 | 0.0556 (1) | 0.0119 (13/1,089) | 0.2062 |
| B*42 | 0 | 0.0040 (5/1,252) | 1.0000 |
| B*44 | 0.1111 (2) | 0.2866 (362/1,263) | 0.1192 |
| B*45 | 0.0556 (1) | 0.0177 (22/1,241) | 0.2841 |
| B*47 | 0 | 0.0039 (2/513) | 1.0000 |
| B*49 | 0 | 0.0171 (21/1,230) | 1.0000 |
| B*50 | 0 | 0.0140 (17/1,212) | 1.0000 |
| B*51 | 0 | 0.0696 (87/1,250) | 0.6291 |
| B*52 | 0 | 0.0180 (21/1,164) | 1.0000 |
| B*56 | 0 | 0.0097 (9/928) | 1.0000 |
| B*57 | 0.0556 (1) | 0.0385 (39/1,014) | 0.5121 |

^a^ Abbreviations: HLA, human leukocyte antigen; IgGSD, immunoglobulin G subclass deficiency.

^b^ Comparisons were made with Fisher's exact test (two-tailed). These are nominal values of *p*. Bonferroni correction for 23 comparisons yielded a revised *p* for significance of <0.0022.
