# Supplementary material for: Characterization of adult patients with IgG subclass deficiency and subnormal IgG2
Source: PLoS One. 2020 Oct 13;15(10):e0240522. doi: 10.1371/journal.pone.0240522 (PMC7553271; doi:10.1371/journal.pone.0240522)
Supplement: S3 Table — (DOCX) [file pone.0240522.s004.docx]

**S3 Table.** HLA-A and -B haplotype frequencies in 17 adults with IgGSD and low IgG2.^a^

| **HLA-A** | **HLA-B** | **Patients (34 chromosomes)** | **Controls (n chromosomes)** | **Value of p**^b^ |
| --- | --- | --- | --- | --- |
| 01 | 08 | 0.1471 (5) | 0.0925 (1502) | 0.2417 |
| 02 | 07 | 0.0588 (2) | 0.0413 (1502) | 0.6499 |
|  | 08 | 0.0294 (1) | 0 (1502) | 0.0221 |
|  | 15 | 0.0294 (1) | 0.0306 (1502) | 1.0000 |
|  | 18 | 0.0294 (1) | 0.0083 (1210) | 0.2637 |
|  | 27 | 0.0294 (1) | 0.0083 (1210) | 0.2637 |
|  | 35 | 0.0294 (1) | 0.0083 (1210) | 0.2637 |
|  | 40 | 0 | 0.0073 (1502) | 1.0000 |
|  | 44 | 0.0588 (2) | 0.0633 (1502) | 1.0000 |
|  | 51 | 0.0294 (1) | 0.0127 (1502) | 0.3627 |
|  | 52 | 0.0294 (1) | 0 (1502) | 0.0221 |
| 03 | 07 | 0.0588 (2) | 0.0546 (1502) | 0.7089 |
|  | 14 | 0.0588 (2) | 0.0113 (1502) | 0.0643 |
|  | 27 | 0.0294 (1) | 0 (1502) | 0.0221 |
|  | 44 | 0 | 0.0127 (1502) | 1.0000 |
|  | 35 | 0.0588 (2) | 0 (1502) | 0.0005 |
| 11 | 8 | 0.0294 (1) | 0 (1502) | 0.0221 |
|  | 13 | 0.0294 (1) | 0 (1502) | 0.0221 |
|  | 51 | 0.0294 (1) | 0.0033 (1210) | 0.1296 |
| 24 | 7 | 0.0294 (1) | 0.0050 (1210) | 0.1767 |
| 29 | 35 | 0.0294 (1) | 0 (1502) | 0.0221 |
|  | 44 | 0.0294 (1) | 0.0233 (1502) | 0.5575 |
|  | 45 | 0.0294 (1) | 0 (1502) | 0.0221 |
|  | 49 | 0.0294 (1) | 0 (1502) | 0.0221 |
| 34 | 8 | 2.9 (1) | 0 (1502) | 0.0221 |

^a^ Abbreviations: HLA, human leukocyte antigen; IgGSD, IgG subclass deficiency; IgG2, immunoglobulin G2. These data represent observations in 17 unrelated non-Hispanic white patients for whom haplotyping observations were available (= 34 chromosomes) and controls (other white adults who underwent haplotype analysis for paternity testing). All subjects were residents of Alabama. All haplotypes could be detected with both DNA-based and serologic methods except haplotypes containing B*70 and B*72 that were not detected by serologic methods. Frequencies are displayed as % (n = chromosomes).

^b^ Comparisons were made with Fisher's exact test (two-tailed). These are nominal values of *p*. Bonferroni correction for 25 comparisons yielded a revised *p* for significance of <0.0020..
